# High-intensity intermittent “5–10–15” running reduces body fat, and increases lean body mass, bone mineral density, and performance in untrained subjects

**DOI:** 10.1007/s00421-018-3851-x

**Published:** 2018-03-29

**Authors:** Tanja Ravnholt, Jonas Tybirk, Niklas Rye Jørgensen, Jens Bangsbo

**Affiliations:** 1 Section of Integrative Physiology, Department of Nutrition, Exercise and Sports, University of Copenhagen, August Krogh Building, Universitetsparken 13, 2. Floor, 2100 Copenhagen Ø, Denmark; 2 Research Center for Ageing and Osteoporosis, Copenhagen University Hospital Glostrup, Copenhagen, Denmark

**Keywords:** Bone markers, Body composition, High-intensity training, Pulmonary oxygen uptake

## Abstract

The present study examined the effect of intense intermittent running with 5 s sprints on body composition, fitness level, and performance in untrained subjects aged 36–53 years. For 7 weeks, the subjects carried out 3 days a week 5–10–15 training consisting of 3–9 blocks of 4 repetitions of 15, 10, and 5 s low-, moderate-, and high-speed running, respectively. Body fat mass was 4.3% lower (*P* < 0.01), and lean body mass and bone mineral density was 1.1 and 0.9% higher (*P* < 0.01), respectively, after compared to before the intervention period (INT). The plasma bone turnover markers osteocalcin increased (*P* < 0.01) by 147%, and procollagen-type I N propeptide and carboxy-terminal collagen crosslinks increased (*P* < 0.05) by 84 and 76%, respectively. Furthermore, the training improved performance in 1500 m (*P* < 0.001), 3 km (*P* < 0.001), Yo–Yo intermittent endurance test (*P* < 0.01), and incremental treadmill running (*P* < 0.001) by 8.1, 9.9, 17.2, and 23.9%, respectively. Furthermore, blood lactate after running at 85% of maximal aerobic speed was lower (*P* < 0.01) after compared to before the INT. Thus, 7 weeks of 5–10–15 training resulted in significant health beneficial changes and better performance in untrained subject.

## Introduction

Regular physical activity improves cardiovascular health profile and performance of untrained individuals, and is associated with changes in body composition, such as lowering of body fat mass and in some instances increase in bone formation, which may reduce prevalence of osteoporosis in later life (LeMura et al. 2000; Scharhag-Rosenberger et al. 2009; Mohr et al. 2007; Krustrup et al. 2010; Nybo et al. 2010). However, the effect of the physical activity depends on the duration, intensity, and frequency of the training sessions. Lack of time is often used as an argument when people fail to accomplish the training programs. Therefore, it is interesting, that exercise at near maximal or maximal intensities effort seems to be effective to create muscle and cardiorespiratory adaptations causing better work capacity in both untrained and trained individuals after just a short period of training (Burgomaster et al. 2005; Gibala et al. 2006; Mohr et al. 2007; Iaia et al. 2008; Gunnarsson and Bangsbo 2012; Gliemann et al. 2014; Skovgaard et al. 2014; Vorup et al. 2016). Thus, as little as six sessions with four to six 30-s all-out intervals, over a 2-week period has been shown to increase maximal citrate synthase activity and endurance capacity in untrained individuals (Burgomaster et al. 2005; Gibala et al. 2006). Even in already trained runners, a 4-week period with training consisting of 30-s maximal bouts improved short-term performance and maintained long-term performance, despite a ~ 65% reduction in training volume compared to before study start (Iaia et al. 2008). Furthermore, reducing training volume 25–50%, and conducting both aerobic high-intensity training, and training consisting of 30-s sprints in a 6–9-week period also improved long-term performance in well-trained subjects (Bangsbo et al. 2009; Skovgaard et al. 2014; Vorup et al. 2016). In addition, training using only 10-s sprints in the 10–20–30 training concept has shown remarkable improvement in maximum oxygen consumption and performance as well as health profile of both untrained and trained subjects, even with a markedly reduced volume of training (Gunnarsson and Bangsbo 2012; Gliemann et al. 2014). In the 10–20–30 training concept, heart rate was above 90% of maximal heart rate for more than 11 min of the 20-min training session. It is, however, unclear whether untrained individuals can benefit from repeated sprints of just 5-s duration, separated by active recovery in between sprints as introduced in the 5–10–15 training concept consisting of 15, 10, and 5 s of low-, moderate-, and high-speed running, respectively.

In untrained subjects, regular aerobic training can lead to reduced body fat (Krauss et al. 2002; Pedersen and Saltin 2006; Nybo et al. 2010; Krustrup et al. 2010). However, the amount of training and intensity seems to be of importance for fat loss and alteration in body composition (Kraus et al. 2002; Nybo et al. 2010; Rosenkilde et al. 2012; Trapp et al. 2008). Taken intensity into consideration, Nybo et al. (2010) compared the effect of intense interval running and prolonged running and found that fat mass was only lowered after prolonged running. In contrast, Trapp et al. (2008) found a decreased fat mass after repeated sprint training but not prolonged cycling, even though the energy expenditure during the 15 weeks of training was equal. Nevertheless, it is unclear whether 5–10–15 training can lead to reduced body fat mass.

Throughout life, it seems to be important to have a frequent load of high impact training to reach the highest possible peak bone mass, and to avoid loss of bone mass, later in life. Mechanical loading when performing exercise is an important stimulus for increase in bone mineral content, bone mineral density, and bone turnover markers (Drinkwater 1994; Heinonen et al. 1995; Helge et al. 2014). Thus, running at moderate speed and even interval running with intensities corresponding to *V*O_2_ max do not provide sufficient stimuli for enhancing bone mass (Nybo et al. 2010). Weight-bearing activities will lead to a high osteogenic impact that can prevent the risk of develop osteoporosis in later life (Bass et al. 1998). Nevertheless, it appears necessary to have high impact from ground reaction forces, and bone adaptations are higher, when breakings, accelerations, and turnings are part of the training (Robling et al. 2002; Kohrt et al. 2009), e.g., sprinting, where the thigh muscles are heavily activated and the bone tissue is exposed to high strain rate on the longitudinal bone axis (Simonsen et al. 1985). It has recently been shown that team sport has a positive effect on bone mineral density in pre- and post-menopausal women as well as elderly men (Seidelin et al. 2017; Helge et al. 2014). These activities are associated with short periods of accelerations, sprinting, breakings, turns, and rapid change in running direction. Thus, it is hypothesized that the 5–10–15 running with the short accelerations, sprinting, and breakings during the 5-s intervals will have a positive effect on bone mineral density.

Thus, the aim of the present study was to examine the effect of training with 5-s sprints followed by low- and moderate-intensity running (5–10–15 concept) on *V*O_2_ max, bone mineral density, bone turnover markers, and body composition. In addition, various running tests were included to obtain a broad spectrum measure of the effect of the training on performance. It was hypothesized that despite the short sprints and low training volume, *V*O_2_ max, performance, and bone turnover would improve, and body fat would decrease in untrained subjects during 7 weeks of training.

## Methods

### Subjects

Eleven untrained subjects (7 women and 4 men) with an average age (ranged 36–53 years), body mass, and *V*O_2_ max of 42.7 ± 1.5 year, 78.4 ± 4.4 kg, and 38.3 ± 1.6 ml/kg/min, respectively, participated in the study. To be included in the study, the adults should be nonsmokers, not having done regular training for the last 2 years and performing less than 2 h of physical activity per week, and not having a *V*O_2_ max above 40 and 45 ml/min/kg for women and men, respectively. During the intervention period, the subjects did not change their daily activities but participated in the 5–10–15 training. The physical characteristics and *V*O_2_ max of the subjects who completed the intervention are presented in Table 1. During the INT, one woman left the study due to injury, a woman withdrew due to disease in the family, and another woman was not included in the data due to injury and, therefore, low compliance to training.

**Table 1:** Characteristics of subjects

	Subjects (*n* = 11)
Age, year	42.7 ± 1.5
Height, cm	175.1 ± 2.4
Body mass, kg	78.4 ± 4.4
*V*O_2_ max, ml/min/kg	37.1 ± 1.4

Values are means ± SE

*n* no. of subjects; *VO*_*2*_ *max* maximal oxygen uptake

All participants were fully informed of experimental procedures and any discomforts associated with participating in the study before signing a written informed consent. The study was approved by the Ethics Committee of Copenhagen and Frederiksberg communities and conformed to the code of the Ethics of the World Medical Association (Declarations of Helsinki) and the Title 45, U.S. Code of Federal Regulations, Part 46, Protection of Human Subjects, Revised November 13, 2001 (Revised June 23, 2005, effective June 23, 2005).

### Experimental design

In a 7-week intervention period (INT), the subjects conducted 5–10–15 training (see *Training*) three times per week. Three weeks prior to the start of the study start subjects, underwent the following familiarization tests to avoid a learning effect when repeating the test after the INT: (1) a treadmill test to determine *V*O_2_ max; the velocity which elicited *V*O_2_ max (maximal aerobic speed, MAS); and time to exhaustion, (2) a 1500-m run, (3) the Yo–Yo Intermittent Endurance test level 1 (Yo–Yo IE1). Before the start and immediately after the end of INT, the aforementioned tests were replicated and a 3-km run was added to the test battery. In addition, on a separate day before and after the INT, subjects reported to the laboratory after an overnight fast to a dual-energy X-ray absorptiometry (DEXA) scan and a fasting blood sample.

### Training

Training was conducted during spring and early summer in a park area on a smooth path of pebble and gravel. Every training session consisted of a standardized 15-min warm-up, including pair exercises and running drills (heel kicks, knee lifts, and side hops) followed by two 100-m runs with acceleration. After the warm-up, subjects did repeated 2-min periods of 5–10–15 running interspersed by 1 min of rest. In the first week, subjects performed three 2-min periods. The total number of 2-min periods increased by 1 every week until subjects completed nine running periods in the final week of the INT. Each 2-min period of 5–10–15 running consisted of 4 consecutive 30-s intervals divided into 15, 10, and 5 s at a running speed corresponding to ~ 30, ~ 50, and ~ 85% of highest maximal speed during training as determined with 5-Hz GPS. In between the 2-min periods, the subjects had a passive recovery for 1 min. The GPS units were placed into the manufacturer-designed harness, which was worn during training. After GPS recording during training, the data were downloaded and analyzed using proprietary software (Sprint, Catapult Sports, Canberra, Australia). Subjects ran for 29 and 15% of the running time with a speed above 85 and 100%, respectively, of the velocity that elicited maximal aerobic speed, respectively. The subjects completed 2.3 ± 0.12 training session per week during INT. The training distance was measured with a 5-Hz GPS system at a minimum of two occasions for each subject within the first 2 weeks of training and the last 2 weeks of training. The average volume of the 5–10–15 running of each subject increased from 2160 ± 115 m/week in the first week to 6479 ± 345 m/week in the final week (warm-up not included) of INT. Subjects were wearing heart rate monitors at least at two training sessions during INT. Heart rate response was related to the calculated maximal heart rate (220—age beats per min) or individual peak heart rate obtained during training.

### Performance measurements

Before and within the first 14 days following the 7-week intervention period, the subjects completed testing with no more than 4 days between the tests. Prior to testing, the subjects refrained from severe physical activity for at least 48 h. The warm-up before conducting the Yo–Yo IE1, 1500-m, and 3-km run consisted of 15 min with low-to-moderate speed running including running drills (heel kicks, knee lifts, and side hops), dynamic stretches and mobilizing exercises. Before each test, the subjects was told to complete the 1500-m and 3-km test as fast as possible and continue as far as possible in the Yo–Yo IE1 test. The subjects had no information about their individual performance before completion of the last test in the study.

#### 1500-m and 3-km

The 1500-m and 3-km run was carried out on a GPS measured 1.5-km course. The subjects did not wear watches during the tests and were not aware about running time. The time to complete the 1500 m and 3 km was used as the test result.

#### Yo–Yo intermittent endurance test level 1

On a separate occasion, the subjects completed the Yo–Yo IE1 test consisting of repeated 20-m shuttle runs, back and forth between the starting line and finish line marked by cones, at a progressively increased running speed dictated by audio bleeps from a pre-recorded source. Between each shuttle, the subjects had a 5-s rest period of slow jogging or walking around a cone placed 2.5 m from the starting line. The test result was recorded as the distance covered at the point when subjects have failed twice to reach the finishing line in between the bleep. All testing sessions were performed indoor on 2 × 20-m running lanes marked by cones (Bangsbo 1994).

#### Submaximal and incremental test

A familiarization test was carried out as an incremental test to exhaustion. The subjects started at a velocity of 5–8 km/h based on individual exercise history. The speed was gradually increased until volitional exhaustion. MAS was determined as the speed, which elicited *V*O_2_ max during the familiarization test. The test was performed on a motorized treadmill under standard laboratory conditions. On a second occasion, the subjects completed a submaximal treadmill running test followed by an incremental test to exhaustion. The subjects arrived at the laboratory, and before start of the test, the subjects had a catheter (18 gauge, 32 mm) inserted in an antecubital vein. The test consisted of two 5-min runs corresponding to 70% of MAS (8.0 ± 0.6 km/h) and 85% of MAS (10.1 ± 0.5 km/h), separated by a 2-min rest period. Submaximal running speeds were estimated from the determination of MAS obtained during the familiarization test. Two subjects walked at 5 km/h, instead of running, as 70% of MAS for these subjects was below 7 km/h. Two minutes after the cessation of the second submaximal bout, subjects carried out an incremental test starting with 1 min at a speed of 85% of maximal aerobic speed. Hereafter, running speed increased by 0.5 km/h every minute until volitional exhaustion. The time to exhaustion was measured from the beginning of the incremental test and until volitional exhaustion, and the time used as the test result. Pulmonary *V*O_2_ was measured throughout the exercise periods by a breath-by-breath gas analyzing system (Oxycon Pro, Viasys Healthcare, Hoechberg, Germany), which was calibrated before each test. *V*O_2_ max was determined as the highest value achieved over a 30-s period. To meet the criteria for achievement of *V*O_2_ max at least two of the following three criteria should be achieved: (1) plateau in *V*O_2_ despite a further increase in speed; (2) a respiratory exchange ratio (RER) > 1.10 and (3) lactate concentration above 10 mmol/l after completing the incremental test to exhaustion. To determine blood lactate concentration during the submaximal and incremental test, blood samples were collected before the first bout, 1 min after each submaximal running bout as well as 30, 90, and 180 s after exhaustion using 2-ml heparinized syringes. Blood was analyzed for lactate concentration using an ABL 800 Flex (Radiometer, Copenhagen, Denmark) immediately after testing.

### DEXA scan and resting blood samples

Subjects reported to the laboratory between 6 and 9 a.m. on a separate day after an overnight fast. Body composition was assessed from whole-body DEXA scanning (Prodigy Bone Densitometer System; GE Lunar, Madison, WI) with measurements of lean body mass, total fat mass, regional fat mass, bone mineral content (BMC), and bone mineral density (BMD) according to standard procedures. It has previously been reported that the precision error of the DEXA scanner is between 0.6 and 1.8% (Shepherd et al. 2006). Fasting blood was taken from the antecubital vein and collected to determine the concentration of bone turnover markers. A total of 1.5 mL of blood collected from a non-heparinized syringe was diluted in 30 mL EDTA and centrifuged for 2 min, after which the plasma was pipetted and frozen at − 80 °C until analyzed for bone markers. Serum/plasma Carboxy-terminal collagen crosslinks (CTX) was measured using the IDS-iSYS CTX (CrossLaps^®^) assay (Immunodiagnostic Systems, plc, Tyne and Wear, UK). Serum/plasma procollagen-type I N propeptide (P1NP) was measured using the IDS-iSYS intact P1NP assay (Immunodiagnostic Systems). Finally, serum/plasma osteocalcin was measured using the N-Mid Osteocalcin assay (Immuodiagnostic Systems). All assays were carried out on a dedicated automated analyzer, iSYS (Immunodiagnostic Systems) according to the manufacturer’s instructions. All three assays are chemiluminescence immunoassays.

All analysis were done with serum/plasma as the sample material. For each assay, the sample aliquots were kept frozen at − 80 °C until the day of analysis. None of the samples had previously been thawed, and all analysis were performed immediately after thawing the samples. All samples were analyzed using one single batch of each assay. Assay performance was verified using the manufacturers’ control specimens. The intermediary precisions expressed as coefficients of variation for CTX were 5.3% (at CTX concentration 213 ng/L), 3.4% (869 ng/L), and 3.5% (2113 ng/L) for iSYS. For P1NP, the intermediary precisions were 5.4% (18.96 µg/L), 6.5% (48.48 µg/L), and 6.1% (122.10 µg/L) for iSYS. Finally, for osteocalcin, the intermediary precisions were 3.0% (8.73 µg/L), 3.6% (27.6 µg/L), and 3.5% (68.7 µg/L). The reference values for Osteocalcin are 10.4–45.6 µg/L; P1NP are 27.7–127.6 µg/L. Furthermore, reference values for CTX are 0.115–0.748 µg/L for men and 0.112–0.738 µg/L in premenopausal women and 0.142–1.351 µg/L in post-menopausal women.

### Statistics

Changes in performance (1500-m, 3-km, and Yo–Yo IE1), pulmonary *V*O_2_, fasting blood bone markers (Osteocalcin, CTX, and P1NP), and blood lactate were evaluated using a two-way analysis of variance for repeated measurement with a linear mixed model approach which was applied to the data using a the *lme4* packages. Corresponding t tests and adjusted *P* values were calculated using the package *multcomp*. Measurements at the end of the intervention were used as dependent variables and measurements at baseline were the independent variables. A significance level of *P* < 0.05 was chosen. Data are presented as means ± standard error (SE). The R (R Core Team) statistic tool was used in all statistical analyses. To evaluate correlation (Pearson correlation) between training volume and changes in performance, pulmonary *V*O_2_, body composition, bone mineral density, blood bone turnover markers, and blood lactate, a Chi-squared distribution test was used.

## Results

### Heart rate during training

**Fig. 1:**
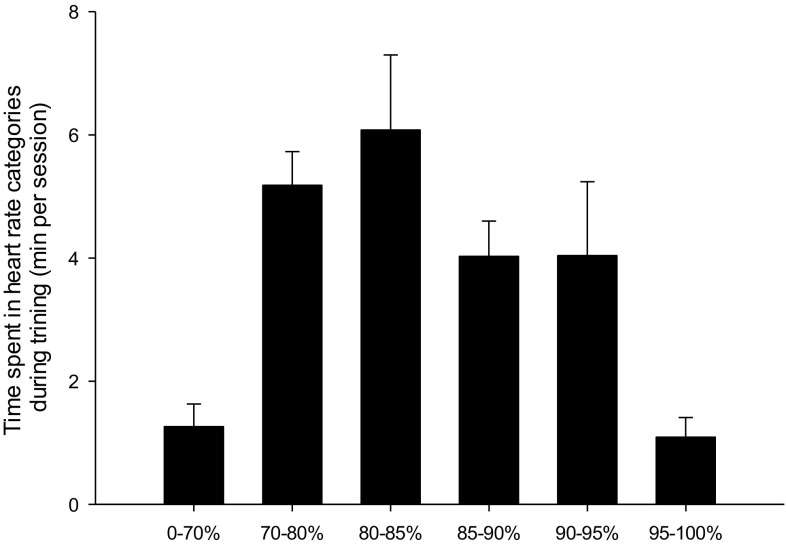
Time spent in various heart rate zones during a 5–10–15 training session

Mean heart rate during a training session was 84 ± 1.2% of maximal heart rate (warm-up not included) and heart rate was above 90% of maximal heart rate for 23% of the session (Fig. 1).

### Body composition

The total body weight was unchanged, but lean body mass was 1.1% higher (*P* < 0.05) after compared to before INT (52.6 ± 3.0 kg vs. 52.0 ± 3.1 kg; Table 2). Body fat, expressed as percentage of total body mass, was lower (*P* < 0.001) after compared to before INT (33.3 ± 2.3 vs. 34.8 ± 2.3%). Furthermore, android and gynoid fat mass was lower (*P* < 0.01) after compared to before the INT (40.3 ± 1.9 vs. 42.1 ± 1.9% and 39.9 ± 2.8 vs. 41.3 ± 2.8%; Table 2).

**Table 2:** Weight, lean body mass, fat mass, android, and gynoid fat mass before (Pre) and after (Post) a 7-week period with 5–10–15 training

	Pre	Post
Weight, kg	78.6 ± 4.6	77.8 ± 4.4
Fat mass%	34.8 ± 2.3	33.3 ± 2.3**
Lean body mass, kg	52.0 ± 3.1	52.6 ± 3.0**
Android fat, %	42.1 ± 1.9	40.3 ± 1.9**
Gynoid fat, %	41.3 ± 2.8	39.9 ± 2.8**

Values are means ± SE

**Different (*P* < 0.01) from Pre

### Bone mineral content, bone mineral density, and bone turnover markers

**Fig. 2:**
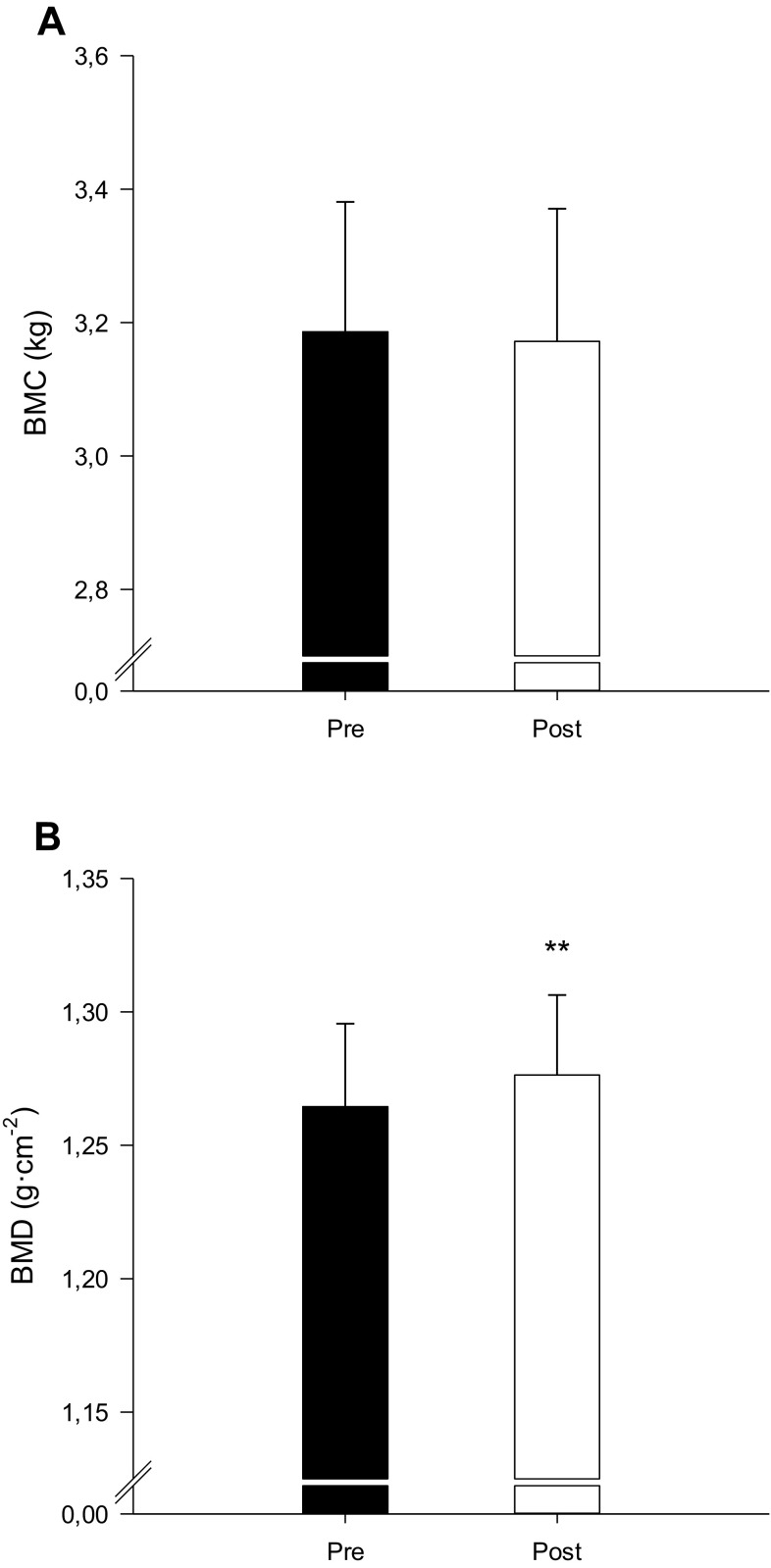
Bone mineral content (BMC; **a**) and bone mineral density (BMD; **b**) before (Pre) and after (Post) a 7-week period with 5–10–15 training. **Different (*P* < 0.01) from Pre

**Fig. 3:**
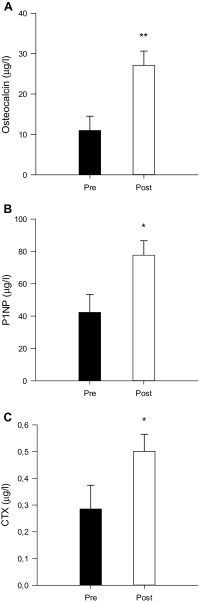
Plasma osteocalcin (A), procollagen-type I N-terminal (P1NP; B), and carboxy-terminal collagen crosslinks (CTX; C) before (Pre) and after (Post) a 7-week period with 5–10–15 training. *Different (*P* < 0.05) from Pre. **Different (*P* < 0.01) from Pre

Whole-body BMD was higher (*P* < 0.01) after compared to before INT (1.276 ± 0.03 vs. 1.265 ± 0.03 g/cm^2^; Fig. 2a), with no changes in whole-body BMC (Fig. 2b). The level of osteocalcin was threefold higher (*P* < 0.01) after compared to before INT (27.1 ± 3.5 vs. 11.0 ± 3.5 µg/L), CTX and P1NP were 76 and 84% higher (*P* < 0.05) after compared to before INT (500 ± 60 vs. 290 ± 90 ng/L and 77.7 ± 9.0 vs. 42.3 ± 11.0 µg/L), respectively (Fig. 3).

### Performance

**Fig. 4:**
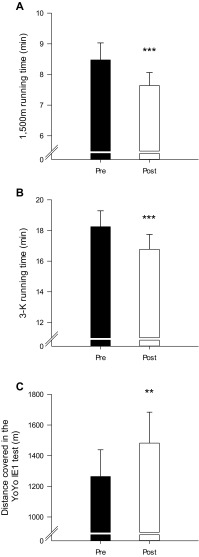
Performance in 1500-m (**a**), 3-K (**b**), and Yo–Yo intermittent endurance (**c**) test before (Pre) and after (Post) a 7-week period with 5–10–15 training. **Different (*P* < 0.01) from Pre. ***Different (*P* < 0.001) from Pre

**Fig. 5:**
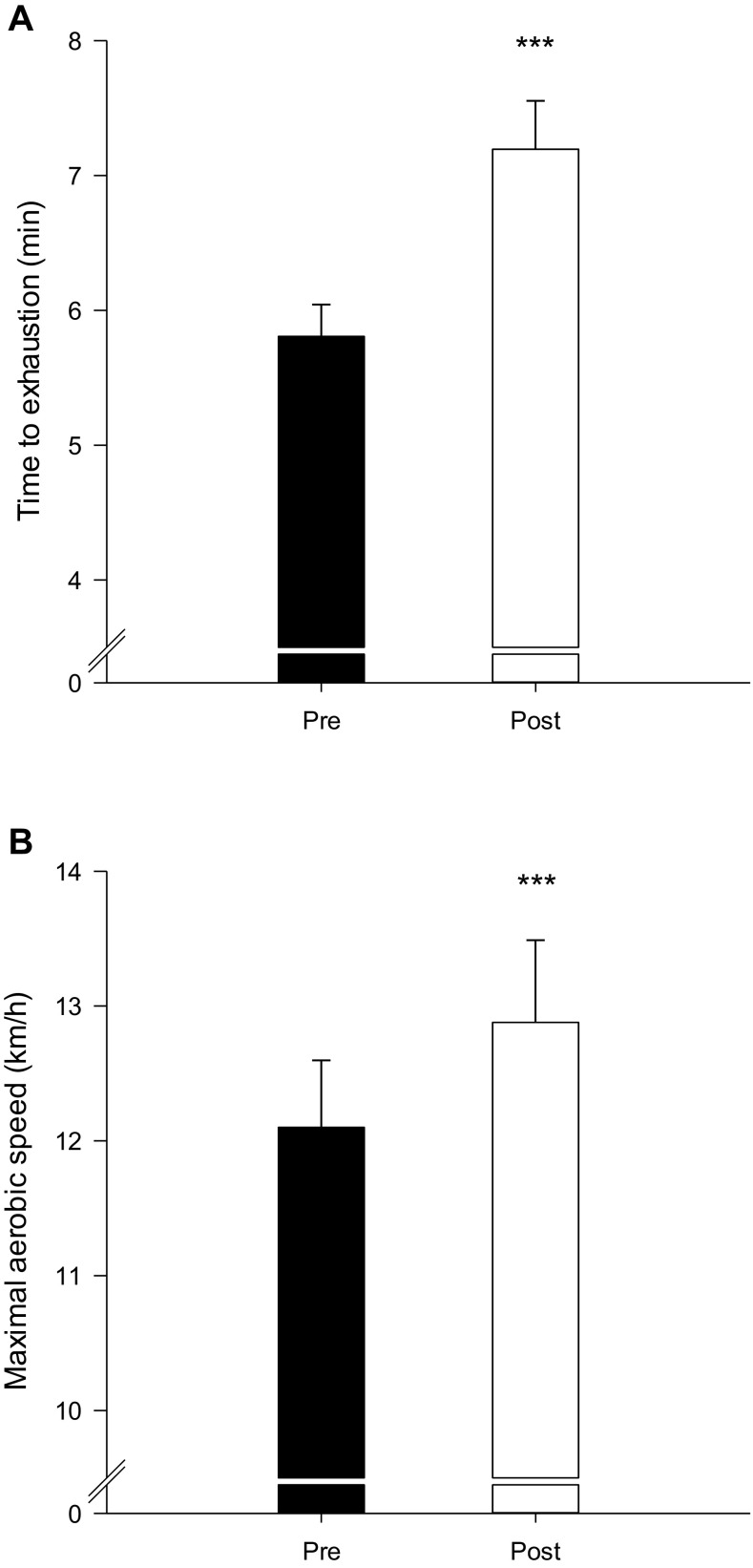
Time to exhaustion (**a**) and maximal aerobic speed (**b**) during a treadmill test before (Pre) and after (Post) a 7-week period with 5–10–15 training. ***Different (*P* < 0.001) from Pre

The time to complete the 1500-m running test was reduced (*P* < 0,001) by 9.9% after compared to before INT (7.64 ± 0.4 vs. 8.48 ± 0.6 min; Fig. 4a), and 3-km running test was 8.1% shorter (*P* < 0.001) after compared to before INT (16.77 ± 1.0 vs. 18.25 ± 1.0 min; Fig. 4B). Distance covered in the Yo–Yo IE1 test was increased (P < 0.01) by 17.2% (1482 ± 202 vs. 1264 ± 174 m; Fig. 4c). The time to exhaustion during the incremental treadmill test was 23.9% longer (*P* < 0.001) after compared to before INT (7.20 ± 0.4 vs. 5.81 ± 0.2 min; Fig. 5a), and the maximal aerobic speed was increased (*P* < 0.001) by 6.4% (12.9 ± 0.6 vs. 12.1 ± 0.05 km/h; Fig. 5b).

### Maximum oxygen uptake

*V*O_2_ max was 5% non-significant higher (*P* = 0.127) after compared to before INT (40.7 ± 1.7 vs. 38.3 ± 1.6 ml/kg/min).

### Blood lactate during treadmill running

The blood lactate concentration after 85% of maximal aerobic speed was 21.1% lower (*P* < 0.01) after compared to before the INT (3.9 ± 0.4 vs. 5.0 ± 0.4 mmol/l) with no other differences in blood lactate during the test (Table 3).

**Table 3:** Blood lactate at rest, after two submaximal running bouts at 70 and 85% of MAS, and after exhaustive treadmill running before (Pre) and after (Post) a 7-week period with 5–10–15 training period

	Running speed			Exhaustion	Recovery	
	Rest	8.0 km/h	10.1 km/h		1 min	3 min
Lactate concentration, mmol/l						
Pre	1.1 ± 0.1	2.7 ± 0.2	5.0 ± 0.4	10.5 ± 0.8	10.6 ± 0.8	10.8 ± 0.8
Post	1.1 ± 0.2	2.4 ± 0.3	3.9 ± 0.4**	11.3 ± 1.1	11.2 ± 1.0	10.9 ± 1.0

Values are means ± SE

**Different (*P* < 0.01) from Pre

## Discussion

The main findings of the present study were that 7 weeks of 5–10–15-training increased bone mineral density and plasma bone turnover markers, as well as improved 1500-m and 3-km running performance. In addition, the 5–10–15-training lowered total fat mass and increased lean body mass. There was no relationship between training volume and change in any of the variables measured.

The 7 weeks of 5–10–15 training lead to higher bone mineral density and elevated the plasma levels of the bone formation markers osteocalcin, CTX, and P1NP, indicating an increased bone formation with the intervention period. This is a promising result concerning the osteogenic effect on bone health of 5–10–15-training. Similarly, after a 4-month period with football training in 65–75-year-old men, levels of plasma osteocalcin and P1NP increased by 45 and 41%, respectively, and after another 8 months of training, CTX was elevated by 43% (Helge et al. 2014). In another study, plasma osteocalcin, P1NP, and CTX increased by 37, 52, and 42%, respectively, when premenopausal women were conducting football training three times a week for 15 weeks (Mohr et al. 2015). Nevertheless, it is remarkable that bone mineral density and plasma bone turnover markers in the present study were elevated considering the activity only averaging 12 min per session and with the subjects performing only 16 training sessions. The improvements in bone health may be due to the intense parts of the training rather than the volume per se. Thus, training volume was only between 2 and 7 km/week, which is significant lower than in other studies (Krustrup et al. 2009; Nybo et al. 2010; Helge et al. 2014). The running speed during the 5–10–15 training was higher than MAS in 14% of the time (1 min and 40 s per session) and led to frequent accelerations and breakings with high mechanical loads causing strain and compression on the bones of the lower limbs (Zamparo et al. 2016). This may have stimulated bone development, since it has been shown that high strain rate and ground reaction forces in varied directions on the longitudinal axis stimulated bone formation (Kohrt et al. 2009; Burr 1997; Robling et al. 2002; Simonsen et al. 1985). Furthermore, physical activity needs to contain weight-bearing elements, be rapidly applied, and being dynamic to have an impact on bone formation (Helge et al. 2014; Heidemann et al. 2013; Mohr et al. 2015). It was probably the sprinting part of the 5–10–15 training that caused the bone changes, since studies using running with moderate intensity did not find alterations in bone formation (Krustrup et al. 2009; Helge et al. 2014). Furthermore, no change was observed in bone mass after 12 weeks of neither interval running nor prolonged running with moderate intensity (80% of maximal heart rate) for 1 h 2.5 times a week (Nybo et al. 2010). Altogether, the present findings demonstrate that 5–10–15-interval running promoted an increase in bone mineral density despite only a short period of training, indicating that the osteogenic response to high ground reaction forces is crucial for bone formation.

The 7-week period with 5–10–15 interval training had significant impact on lean body mass in the untrained subjects. In accordance, soccer training conducted as small-sided games for untrained women 1.8 times a week for 16 weeks and for untrained men 2.3 times a week for 12 weeks resulted in increased lean body mass (Krustrup et al. 2009). On the other hand, running 2.5 times a week at an average intensity corresponding to 82% of maximum heart rate did not change lean body mass (Krustrup et al. 2009). Similarly, a study with untrained men conducting 12 weeks of interval running, consisting of five 2-min intervals at an intensity slightly below MAS, twice a week, did not change lean body mass (Nybo et al. 2010). Apparently, the 5-s sprinting in the 5–10–15 training does lead to elevated muscle mass in untrained men and women. In addition, the 5–10–15 training reduced the fat mass and regional fat mass in the adults, despite the training volume being only ~ 3 km per training session. In accordance with the present findings, it has been shown that a training intervention using repeated 8-s sprints for a 15-week period elicited a 2.5-kg decrease in total body fat, whereas moderate-intensity training failed to reduce fat mass in untrained women (Trapp et al. 2008). Similarly, Nybo et al. (2010) found that in untrained men fat mass was unchanged after 12 weeks of interval running with speeds below MAS, indicating that the high speeds are important for the loss of fat mass when doing interval training.

The subjects had a non-significant 5% increase in *V*O_2_ max during the intervention period. Similarly, Gunnarsson & Bangsbo (2012) found a 4% higher *V*O_2_ max in recreational runners with 10–20–30 training three times a week for 7 weeks, even though the training volume was lowered by as much as 54% during the training period compared to before study start. In the present study and the study by Gunnarsson and Bangsbo (2012), heart rate was above 90% of maximum heart rate for ~ 25% and ~ 40% of the training time, respectively. Thus, these types of intermittent exercises appear to have a significant impact on the heart, which likely has contributed to the higher *V*O_2_ max, although not significant in the present study, which may be due to the limited number of subjects. In general, the amount of high-intense training seems important for change in the maximal oxygen uptake. In trained runners, Helgerud et al. (2007) found that 4 × 4 min high-intense training (heart rate > 90% of maximum heart rate) three times per week for 8 weeks improved *V*O_2_ max, whereas moderate-intensity training did not. The higher *V*O_2_ max does not seem to be due to the 5-s sprinting periods per se, since maximal intensity exercise for 6 s separated by a long recovery (1 min) did not improve *V*O_2_ max in untrained subjects (Mohr et al. 2007). Likewise, no change in *V*O_2_ max was observed in moderately to well-trained runners when performing near maximal running for 30 s followed by 3-min recovery periods (Iaia et al. 2008; Bangsbo et al. 2009; Skovgaard et al. 2014).

The subjects had an 8 and 10% improvement in the 3-km and 1500-m run, respectively. Similarly, Gunnarsson and Bangsbo (2012) observed that 10–20–30 training of trained subjects for 7 weeks resulted in a 6 and 4% better performance on 1500-m and 5-km, respectively. Since the time to exhaustion at the incremental treadmill test was improved without a significant change in *V*O_2_ max, it could be speculated that the anaerobic capacity was elevated with the training intervention, which may have contributed to the better performance in the 1500-m and 3-km runs as well as in the Yo–Yo IE1. In addition, blood lactate was lowered after the submaximal running after compare to before INT, which indicate an improved endurance capacity, as the lactate threshold occurs at a higher percentage of *V*O_2_ max (Coyle et al. 1988; Joyner and Coyle 2008).

In summary, a 7-week period of 5–10–15 training of untrained adults improved performance, increased bone mineral density, and plasma bone turnover markers as well as lean body mass and reduced fat mass.

## Perspectives

Considering the great impact the 5–10–15 training had on performance and body composition, and that it can be carried out in less than 20 min per session, it is an attractive form of training. Other advantages are that the training can be performed in groups of people with different running experience and physical capacity.

## Abbreviations

BMC: Bone mineral content
BMD: Bone mineral density
CTX: Carboxy-terminal collagen crosslinks
INT: Intervention period
MAS: Maximal aerobic speed
P1NP: Procollagen-type I N propeptide
*V*O_2_: Oxygen uptake
*V*O_2_ max: Maximal oxygen uptake
Yo–Yo IE1: Yo–Yo intermittent endurance test level 1
